# Therapien des Morbus Bowen: Systematisches Review und Netzwerk‐Metaanalyse randomisierter kontrollierter Studien

**DOI:** 10.1111/ddg.15866_g

**Published:** 2025-11-14

**Authors:** Yannick Foerster, Kristine Mayer, Marta Dechant, Julia Palaras, Tilo Biedermann, Oana‐Diana Persa

**Affiliations:** ^1^ Klinik und Poliklinik für Dermatologie und Allergologie am Biederstein Technische Universität München

**Keywords:** Bowen, Heilungsrate, Plattenepithelkarzinom in situ, Therapieoptionen, Bowen, clearance rate, Squamous cell carcinoma in situ, therapy options

## Abstract

Morbus Bowen (MB) ist eine intraepidermale bösartige Neubildung, die zu einem invasiven Plattenepithelkarzinom der Haut führen kann. Bisher existiert keine Studie, die Therapieoptionen aus randomisierten kontrollierten Studien (RCT) miteinander verglichen hat. In dieser Arbeit wurden Daten aus verfügbaren RCT extrahiert, um läsionsspezifische Heilungsraten und kosmetische Ergebnisse mit Placebo zu vergleichen.

Wir durchsuchten MEDLINE, EMBASE, CENTRAL und Studienregister bis 30. September 2024, und schlossen neun Studien mit 672 Patienten und 844 Läsionen ein. Die Therapieoptionen, welche mithilfe einer Netzwerk‐Metaanalyse verglichen wurden, umfassten: Exzision, Imiquimod, photodynamische Therapie (PDT), Laserablation (LA), lasergestützte PDT (LA‐PDT), Kryotherapie und 5‐Fluoruracil (5‐FU). Das Verzerrungsrisiko wurde mithilfe des überarbeiteten *Cochrane risk‐of‐bias Tool* evaluiert. Die Studie wurde in PROSPERO registriert (CRD42024583966).

Alle Therapieoptionen zeigten bessere läsionsspezifische Heilungsraten als Placebo. Bezüglich der initialen Heilungsrate zeigte LA‐PDT die besten Ergebnisse, gefolgt von Exzision und LA. Die Exzision zeigte die besten Ergebnisse bezüglich der langfristigen Heilungsrate, gefolgt von LA‐PDT und PDT. LA‐PDT und 5‐FU hatten das beste, die Exzision das schlechteste kosmetische Ergebnis. Limitationen umfassen ein moderates bis hohes Verzerrungsrisiko sowie Heterogenität der Einzelstudien.

Unsere Ergebnisse unterstützen die klinische Entscheidungsfindung, wobei sich LA‐PDT und PDT als effektive Optionen bei guten kosmetischen Ergebnissen erwiesen haben. Die Exzision zeigte jedoch die besten langfrisigen Heilungsraten.

## EINLEITUNG

Beim Morbus Bowen (MB), auch bekannt als kutanes Plattenepithelkarzinom (cSCC) in situ, handelt es sich um eine häufige nichtmelanozytäre intraepidermale bösartige Neubildung. Dieser zeigt sich typischerweise als scharf begrenzte, langsam wachsende erythematöse Plaque und wird häufig als Ekzem oder Psoriasis fehldiagnostiziert.^1^ Neuere Daten aus einem landesweiten Register der Niederlande zeigen eine Verdopplung der Krankheitsfälle zwischen 2005 und 2015.^2^ Der Erkrankungsgipfel liegt in der siebten Lebensdekade und betrifft häufig sonnenexponierte Areale,^2^ insbesondere die Kopf‐ und Halsregion.^3^ Das Risiko einer Progression zu einem invasiven Tumor wird auf circa 3% beziffert.^4^, ^5^ Leider ist bisher nicht vorherzusagen, welcher MB hierzu neigt, weshalb von nationalen und internationalen Leitlinien generell eine frühe und konsequente Therapie empfohlen wird.^6^, ^7^ Hierzu stehen verschiedene topische und interventionelle Therapieoptionen zur Verfügung. Diese beinhalten 5‐Fluoruracil (5‐FU), Imiquimod, photodynamische Therapie (PDT), Laserablation (LA), lasergestützte PDT (LA‐PDT), Kryotherapie und Exzision. Die aktuelle Studienlandschaft bezüglich den entsprechenden Therapieoptionen ist sehr limitiert. Die meisten randomisierten kontrollierten Studien (RCT) und verfügbaren Metaanalysen fokussieren sich primär auf einen direkten Vergleich zwischen PDT und anderen topischen Therapieoptionen, wobei eine direkte Vergleichbarkeit unterschiedlicher Therapieoptionen nicht möglich ist.^8^, ^9^, ^10^, ^11^, ^12^, ^13^ Weitere verfügbare Übersichtsarbeiten beinhalten außerdem nichtrandomisierte Studien, weshalb das Evidenzlevel hier nur als gering bewertet werden kann. Zusammenfassend stehen bisher keine Daten zur Verfügung, um die Effektivität der verfügbaren Therapieoptionen objektiv bewerten zu können, weshalb die gängigen Empfehlungen hauptsächlich auf einem Expertenkonsensus beruhen. Um diese Lücke zu schließen, führten wir eine Netzwerk‐Metaanalyse (NMA) durch, wobei es sich um eine Methode handelt, welche die traditionelle Metaanalyse erweitert, indem sie den Vergleich mehrerer Therapieoptionen relativ zu einer Referenztherapie ermöglicht. Dies geschieht durch Integration sowohl direkter Vergleiche aus verfügbaren Studien (Therapie A vs. Therapie B) als auch indirekter Vergleiche über gemeinsame Interventionen (Therapie A vs. Therapie B in Studie 1 und Therapie B und Therapie C in Studie 2), wobei ein indirekter Vergleich der Therapien A und C über die gemeinsame Vergleichsintervention B möglich ist. Diese Methode erhält die Randomisierung aufrecht und kombiniert die kleinen Gruppengrößen der Einzelstudien, um die statistische Power der Analyse zur erhöhen.^14^, ^15^, ^16^, ^17^ Um die höchstmögliche Evidenz zu gewährleisten, inkludierten wir nur RCT. Alle Therapieoptionen wurden mit Placebo als Referenztherapie verglichen und mithilfe des p‐Scores, welcher sich aus der NMA ergibt, hinsichtlich ihrer Effektivität bewertet.^18^ Die primären Endpunkte umfassten die läsionsspezifische initiale und langfristige Heilungsrate sowie das kosmetische Ergebnis. Als sekundären Endpunkt untersuchten wir zudem die Häufigkeiten unerwünschter Therapienebenwirkungen.

## MATERIAL UND METHODIK

### Studienprotokoll und Registrierung

Wir registrierten unsere Studie im internationalen prospektiven Register für systematische Übersichtsarbeiten (PROSPERO) vor der Datenextraktion (CRD42024583966). Die Studie wurde in Übereinstimmung mit den PRISMA‐Richtlinien für systematische Übersichtsarbeiten und Metaanalysen, einschließlich der Erweiterung für NMA,^19^ dem Cochrane‐Handbuch für systematische Übersichtsarbeiten^20^ sowie der ENTREQ‐Erklärung zur transparenten Darstellung qualitativer Ergebnissynthesen durchgeführt.

### Einschlusskriterien

Unsere Einschlusskriterien wurden gemäß dem PICO‐Schema festgelegt.^21^

*Patienten (* ***P*** *opulation)*: Patienten mit histologischer Diagnose eines MB wurden eingeschlossen.

*Therapieoptionen (* ***I*** *ntervention)*: Alle Patienten mussten mit einer der folgenden Therapieoptionen behandelt worden sein: 5% 5‐FU, Imiquimod, PDT, LA, LA‐PDT, Kryotherapie oder Exzision. Zur besseren Vergleichbarkeit und um kleine und unverbundene Subnetzwerke zu vermeiden, wurde nicht zwischen PDT mit Aminolävulinsäure (ALA‐PDT) und Methylaminolävulinsäure (MAL‐PDT) unterschieden sowie verschiedene Strahlungsquellen zusammengefasst. Verschiedene ablative Lasersysteme (Er:YAG‐ and CO_2_‐Laser) wurden ebenso zusammengefasst. Sequenz‐ oder Kombinationstherapien wurden ausgeschlossen.

*Vergleichsintervention (* ***C*** *omparison)*: Alle Therapieoptionen wurden mit Placebo als Referenzstandard verglichen.

*Endpunkte (* ***O*** *utcomes)*: Die folgenden primären Endpunkte wurden untersucht: *(1)* Läsionsspezifische initiale Heilungsrate, berichtet zwischen 1–3 Monate nach Therapie; *(2)* läsionsspezifische langfristige Heilungsrate, berichtet zwischen 6‐12 Monate nach Therapie; und *(3)* kosmetisches Ergebnis. Der sekundäre Endpunkt umfasste die Sicherheit, definiert als Anteil an Patienten, die eine unerwünschte Therapienebenwirkung erlitten.

### Endpunkte

Für MB existieren keine standardisierten Ergebnismessgrößen für RCT. Wir inkludierten nur Studien, die eine läsionsspezifische Heilungsrate berichteten. Endpunkte *(1)* und *(2)* waren dichotom. Die Heilungsraten wurden klinisch oder histologisch bewertet. Für Endpunkt *(3)* wurden numerische Skalen von 1–4 berichtet. Zur besseren Vergleichbarkeit wurde 1–2 als schlecht/moderat und 3–4 als gut/exzellent definiert. Alle Effektstärken wurden mit Placebo als Referenz verglichen und als Risikoverhältnis (RR) mit 95%‐Konfidenzintervall (95%‐KI) ausgedrückt. Aufgrund unvollständig berichteter Daten, konnte bezüglich des sekundären Endpunktes kein Effektschätzer berechnet werden. Deshalb wurden die absoluten Häufigkeiten unerwünschter Therapieeffekte zusammengefasst. Die Analyse erfolgte wenn möglich in der ursprünglichen Behandlungsgruppe (Intention‐to‐treat).

### Datenbanken und Suchstrategie

Es erfolgte eine systematische Suche in den elektronischen Datenbanken MEDLINE, EMBASE und Cochrane Central Register of Controlled Trials (CENTRAL) von Datenbankgründung bis einschließlich 30. September 2024. Die Suchstrategie ist verfügbar in der Tabelle S1 (Online‐Supplement). Zusätzlich erfolgte eine manuelle Suche für den Suchbegriff „Bowen“ in folgenden klinischen Studienregistern (letzter Zugriff am 30. September 2024): EU Clinical Trials Register (https://www.clinicaltrialsregister.eu), Australian New Zealand Clinical Trials Registry (https://www.anzctr.org.au), US National Institute of Health Clinical Trial Register (https://clinicaltrials.gov).

### Studienauswahl und Datengewinnung

Zwei Untersucher (Y.F. und O.P.) sichteten mithilfe von Ovid, einer webbasierten Suchplattform, verfügbare Studien zunächst anhand von Titel und Abstract und anschließend anhand des Volltexts in Bezug auf die definierten Einschlusskriterien. Die selben Untersucher durchsuchten ebenfalls die klinischen Studienregister. Diskrepanzen wurden durch Konsesus beigelegt. Eine der Studien beinhaltete außerdem Patienten mit aktinischen Keratosen (AK).^22^ Die Daten für die Untergruppe von Patienten mit MB wurden hier einzeln extrahiert. Nur RCT wurden eingeschlossen. Es wurden keine Sprachrestriktionen festgelegt. Es wurden zweiarmige und mehrarmige Studien eingeschlossen.

### Ergebnissynthese

Wir führten eine NMA durch, um Schlussfolgerungen bezüglich der relativen Wirksamkeit mehrerer Therapieoptionen zu ziehen. Hierzu wurde das frequentistische NMA‐Paket netmeta (Version 3.1‐1) für RStudio (Version 4.4.2) verwendet. Aufgrund der Heterogenität der Einzelstudien wurde ein Random‐Effects‐Modell verwendet.^14^ Mithilfe der Funktion pairwise wurde der Arm‐basierte Datensatz in ein kontrastbasiertes Format überführt, welcher das korrekte Format für die Durchführung der NMA mit dem Paket netmeta darstellt.^15^ Placebo wurde als Referenzintervention definiert. p‐Scores wurden mithilfe der Funktion netrank generiert.^18^ Der p‐Score basiert auf Punktschätzern und Standardfehlern des Netzwerkes und ist ein statistisches Maß dafür, dass eine Intervention einer anderen überlegen ist. Forest‐Plots und Netzwerkdiagramme wurden mithilfe der Funktionen forest und netgraph generiert.^23^ Die Heterogenität zwischen den einzelnen Studien wurde mithilfe der I^2^ und τ^2^ Statistik quantifiziert.^24^ Um eine mögliche Inkonsistenz zwischen direkter und indirekter Evidenz des Netzwerkes zu untersuchen, wurde mit der Funktion netsplit die direkte und indirekte Evidenz extrahiert und mittels Node‐Splitting‐Methode verglichen.^25^

### Beurteilung des Verzerrungsrisikos und Bewertung der Evidenzgrade

Ein möglicher Verzerrungseffekt durch die einseitige Veröffentlichung positiver Studien (Publikationsbias) wurde grafisch mittels Funnelplot untersucht, welcher die zentralen Effektschätzer der Vergleiche ihren Standardfehlern gegenüberstellt. Hierbei deutet eine asymmetrische Verteilung auf einen Publikationsbias hin. Die Symmetrie kann zudem mithilfe des Egger‐Tests statistisch bewertet werden.^26^ Zwei Untersucher (Y.F. and O.P.) bewerteten jeweils unabhängig das Verzerrungsrisiko für alle Endpunkte der eingeschlossenen Studien mithilfe des überarbeiteten *Cochrane Risk‐of‐Bias‐Tools* (RoB2).^27^ Das Tool bietet eine Beurteilung der insgesamt auftretenden Verzerrung aus folgenden fünf Kategorien und erstellt farblich codierte Verzerrungsrisiko‐Diagramme: Randomisierungsprozess, Abweichung von der beabsichtigen Intervention, fehlende Daten, Messung des Endpunktes und Auswahl der berichteten Ergebnisse. Die Zuverlässigkeit der Evidenz wurde unter Verwendung der GRADE‐Leitlinien bewertet.^28^, ^29^ Schlussfolgerungen aus der NMA wurden unter Anwendung des minimal kontextualisierten Rahmenansatzes gezogen.^30^

## ERGEBNISSE

### Studienauswahl und Studiencharakteristiken

**ABBILDUNG 1:**
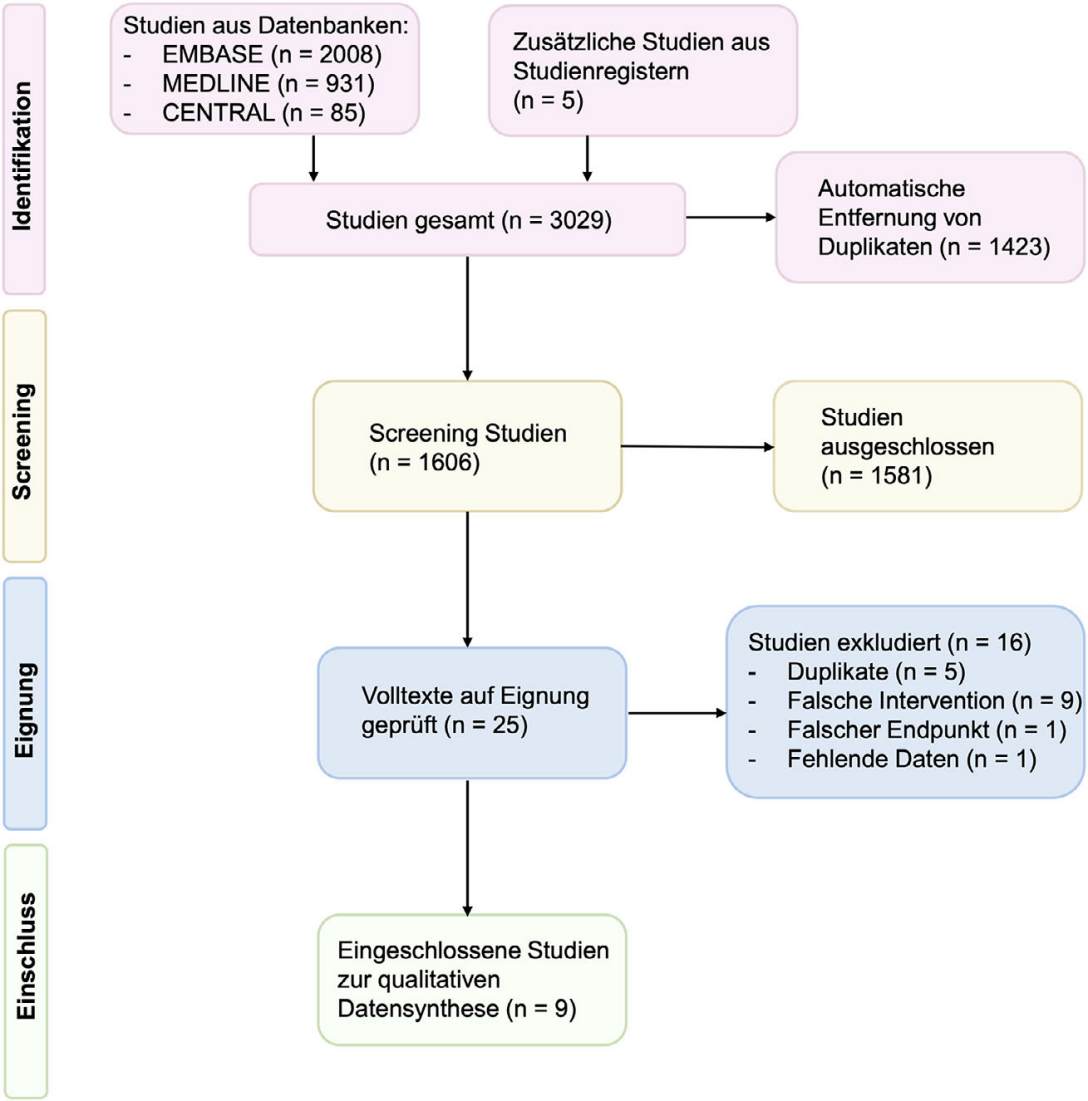
Flussdiagramm des Auswahlprozesses nach den PRISMA‐Vorgaben für systematische Übersichtsarbeiten und Metaanalysen, einschließlich der Erweiterung für Netzwerk‐Metaanalysen (NMA).

Unsere systematische Literaturrecherche identifizierte 3029 Studien. Nach dem automatischen Ausschluss der Duplikate verblieben 1606 Studien. Von diesen wurden 1581 weitere Studien nach Sichtung von Titel und Abstract exkludiert. Insgesamt wurden 25 Volltexte gesichtet, und schließlich neun Studien, die 672 Patienten und 844 Läsionen umfassten, zur qualitativen Datensynthese inkludiert.^22^, ^31^, ^32^, ^33^, ^34^, ^35^, ^36^, ^37^, ^38^ Der Ausschluss der anderen Studien erfolgte aufgrund einer falschen Intervention (n = 9), eines falschen Endpunktes (n = 1) oder aufgrund fehlender Daten (n = 1).^39^, ^40^, ^41^, ^42^, ^43^, ^44^, ^45^, ^46^ Zusätzlich wurden fünf weitere Duplikate identifiziert und manuell exkludiert (Abbildung 1). Alle exkludierten Studien sowie eine kurze Beschreibung sind in der Tabelle S2 (Online‐Supplement) aufgeführt.

Bis auf eine Studie, welche organtransplantierte Patienten untersuchte, schlossen alle Studien immunkompetente Patienten ein.^22^ Eine Studie beinhaltete außerdem Patienten mit AK, sodass die Daten für MB manuell extrahiert werden mussten.^22^ Von den neun Studien waren sieben Studien zweiarmig,^22^, ^32^, ^33^, ^34^, ^36^, ^37^, ^38^ eine Studie dreiarmig,^31^ und eine Studie vierarmig.^35^ Alle Studien berichteten läsionsspezifische initiale Heilungsraten innerhalb von 1–3 Monaten nach Therapieende. Die läsionsspezifische langfristige Heilungsrate wurde 6–12 Monate nach Therapieende untersucht. Nur vier Studien berichteten das kosmetische Ergebnis.^22^, ^31^, ^34^, ^35^

Von den zweiarmigen Sudien untersuchten zwei Studien PDT gegenüber LA‐PDT,^33^, ^34^ und zwei Studien PDT gegenüber 5‐FU.^22^, ^38^ Die verbleibenden zweiarmigen Studien untersuchten LA gegenüber LA‐PDT,^32^ PDT gegenüber Kryotherapie,^36^ und Imiquimod gegenüber Placebo.^37^ Eine dreiarmige Studie untersuchte die Exzision gegenüber 5‐FU und PDT.^31^ Die vierarmige Studie untersuchte Kryotherapie, 5‐FU und PDT gegenüber Placebo.^35^ Tabelle 1 gibt einen detaillierten Überblick über alle eingeschlossenen Studien.

**TABELLE 1:** Überblick über die eingeschlossenen Studien.

					Geschlecht									Berichtete Endpunkte					
Autor	Jahr	Land	Patienten	Durchschnittsalter (Spannweite)	Männlich (%)	Weiblich (%)	Anzahl Läsionen	Durchmesser der Läsionen (mm)	Intervention 1	Intervention 2	Intervention 3	Intervention 4	Dauer des Follow‐up (Monate)	Initiale Heilungsrate (Monate nach Therapie)	Langfristige Heilungsrate (Monate nach Therapie)	Kosmetisches Ergebnis	UAW	Fallzahlberechnung (Power)	Kommentar
Ahmady et al.	2024	Niederlande	250	76 (51–94)	92 (36.8)	158 (63.2)	250	4–40 (Spannweite)	Exzision (5mm Sicherheitsabstand)	5% 5‐FU (2 x täglich 4 Wochen)	MAL‐PDT (2 Zyklen)	/	12	Ja (3)	Ja (12)	Ja	Ja	Ja (0.8)	
Cai et al.	2015	China	18	52 (35–72)	8 (44.4)	10 (55.6)	22	26 (Mittelwert)	CO_2_‐Laser (bis 3 Zyklen abhängig von Ansprechen)	CO2‐Laser gestützte ALA‐PDT (bis 3 Zyklen abhängig von Ansprechen)	/	/	6	Ja (1)	Ja (6)	Nein	Ja	NB	
Kim et al.	2018	Südkorea	60	72 (NB)	24 (40.0)	36 (60.0)	84	≥20 (Spannweite)	MAL‐PDT (2 Zyklen)	Erbium:YAG‐Laser gestützte MAL‐PDT (1 Zyklus)	/	/	60	Ja (3)	Ja (12)	Nein	Ja	NB	
Ko et al	2014	Südkorea	21	69 (35–88)	10 (47.6)	11 (52.4)	58	NB	MAL‐PDT (2 Zyklen)	Erbium:YAG‐Laser gestützte MAL‐PDT (1 Zyklus)	/	/	12	Ja (3)	Ja (12)	Ja	Ja	NB	
Morton et al.	2006	Vereinigtes Königreich	225	74 (39–99)	NB	NB	275	6–40 (Spannweite)	Placebo	Kryotherapie (1 Zyklus)	5% 5‐FU (1 x täglich in der ersten Woche, anschließend 2x täglich für 3 Wochen)	MAL‐PDT (2 Zyklen)	12	Ja (3)	Ja (12)	Ja	Ja	Ja (0.9)	
Morton et al.	1996	Vereinigtes Königreich	19	76 (62–88)	NB	NB	40	≤ 21 (Spannweite)	ALA‐PDT (bis 2 Zyklen abhängig von Ansprechen)	Kryotherapie (bis 3 Zyklen abhängig von Ansprechen)	/	/	12	Ja (2)	Ja (6)	Nein	Nein	NB	
Patel et al.	2006	Vereinigtes Königreich	31	74 (54–86)	11 (35.5)	20 (64.5)	31	4.8–42.1 (Spannweite)	Placebo	5% Imiquimod (1 x täglich 16 Wochen)	/	/	9	Ja (3)	Ja (9)	Nein	Nein	Ja (0.9)	
Perrett et al.	2007	Vereinigtes Königreich	8	59 (46–71)	6 (75.0)	2 (25.0)	18	NB	MAL‐PDT (2 Zyklen)	5% 5‐FU (2x täglich 3 Wochen)	/	/	6	Ja (3)	Ja (6)	Ja	Ja	NB	Organtransplantierte Patienten
Salim et al.	2003	Vereinigtes Königreich	40	76 (65–88)	32 (80.0)	8 (20.0)	66	5–40 (Spannweite)	ALA‐PDT (bis 2 Zyklen abhängig von Ansprechen)	5% 5‐FU (1 x täglich in der ersten Woche, anschließend 2 x täglich für 3 Wochen)	/	/	12	Ja (3)	Ja (12)	Nein	Ja	NB	

*Abk*.: UAW, unerwünschte Therapienebenwirkung; 5‐FU, 5‐Fluoruracil; MAL‐PDT, photodynamische Therapie mit Methylaminolävulinsäure; ALA‐PDT, photodynamische Therapie mit Aminolävulinsäure; NB, nicht berichtet

### Beurteilung des Verzerrungsrisikos

**ABBILDUNG 2:**
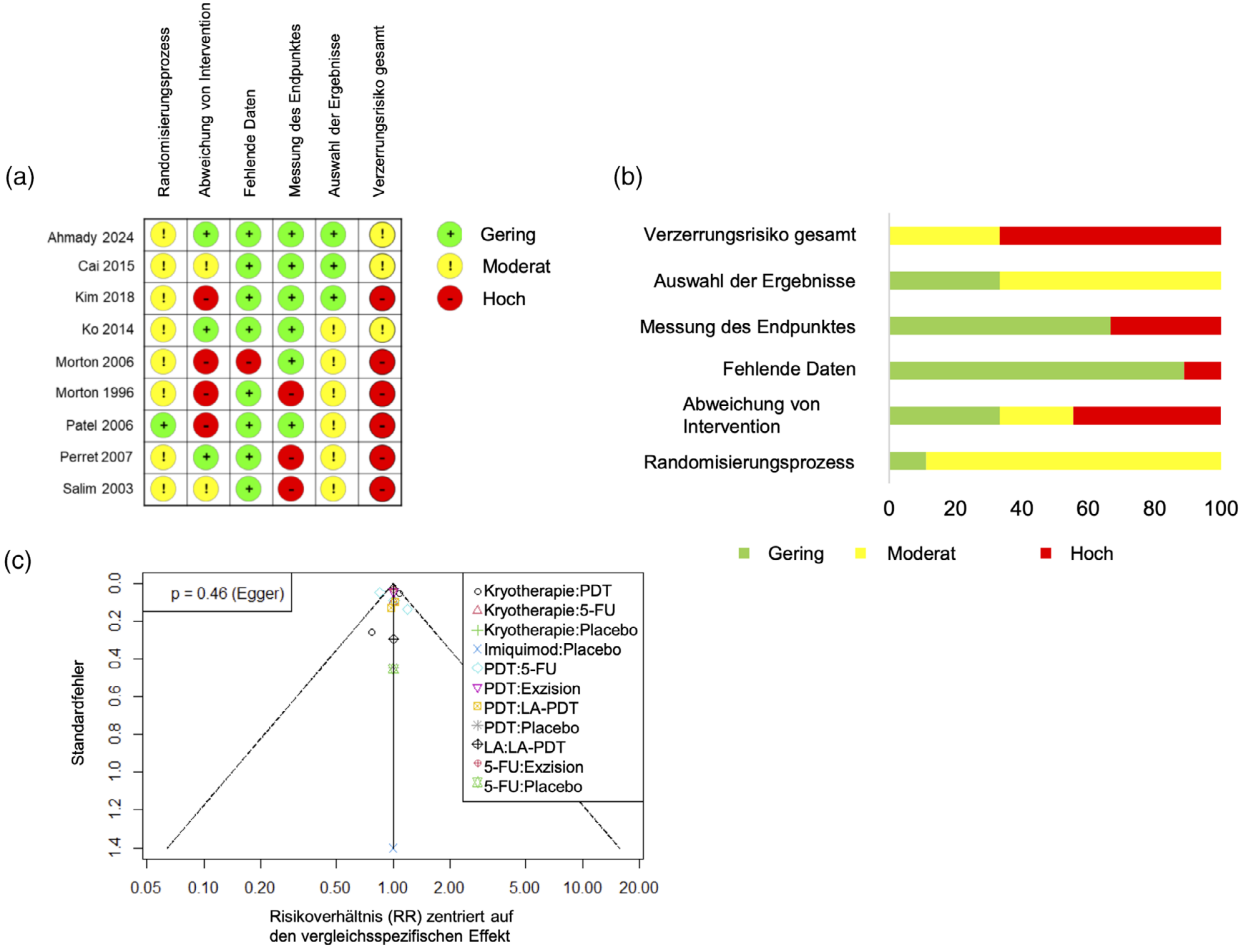
(a) Das Verzerrungsrisiko wurde von zwei unabhängigen Autoren mit Hilfe des überarbeiteten Cochrane Risk‐of‐bias‐Tools für randomisierte kontrollierte Studien (RoB2) bewertet. Das Verzerrungsrisiko für die jeweiligen Katgorien wurde als gering, moderat oder hoch eingesuft. (b) Der Ampel‐Plot zeigt den Anteil an Studien mit niedrigem, moderatem und hohem Verzerrungsrisiko in den jeweiligen Kategorien. Die hier dargestellte Abbildung zeigt die Bewertung der Endpunkte initiale und langfristige Heilungsrate. (c) Im Funnelplot zeigen sich keine Hinweise auf eine Asymmetrie, welche auf einen potenziellen Publikationsbias hindeuten könnte.

Bezüglich der primären Endpunkte initiale und langfristige Heilungsrate war das Verzerrungsrisiko insgesamt als moderat für drei Studien und als hoch für die anderen sechs Studien einzustufen (Abbildung 2a,b). Nur drei Studien gaben an, wie die zufällige Gruppenzuteilung erzielt wurde.^31^, ^33^ Nur eine Studie gab an, dass die Zuteilung verdeckt erfolgte.^37^ Insgesamt war somit bei acht von neun Studien der Randomisierungsprozess nach RoB2 mit einem zumindest moderaten Verzerrungsrisiko behaftet, während eine einzige Studie hierfür nur ein niedriges Verzerrungsrisiko aufwies.^37^ Drei Studien wiesen nur ein geringes Verzerrungsrisiko bezüglich Abweichungen von der beabsichtigten Intervention auf, was bedeutet, dass sowohl Untersucher als auch Patienten adäquat verblinded waren und eine Auswertung eindeutig in der ursprünglich zugeteilten Gruppe erfolgte (Intention‐to‐treat‐Analyse).^22^, ^31^, ^34^ Von den verbleibenden zwei Studien wiesen zwei ein moderates^32^, ^38^ und vier Studien ein hohes Verzerrungsrisiko auf.^33^, ^35^, ^36^, ^37^ Die primären Endpunkte wurden generell gut dargestellt. Nur eine Studie hatte ein hohes Risiko für fehlende Daten,^35^ während die anderen Studien nur ein geringes Risiko aufwiesen. Bezüglich der Messung des Endpunktes wurde das Verzerrungsrisiko für sechs Studien als gering und für die anderen drei Studien als hoch eingestuft.^22^, ^36^, ^38^ Drei Studien wiesen ein geringes Risiko für die Auswahl der berichteten Ergebnisse auf,^31^, ^32^, ^33^ während das Verzerrungsrisiko der restlichen Studien hierfür zumindest als moderat eingestuft werden musste. Bezüglich des kosmetischen Ergebnisses war das Verzerrungsrisiko insgesamt für zwei Studien als hoch und für zwei Studien als moderat einzustufen (Abbildung S1, Online‐Supplement).

Im vergleichsadjustierten Funnelplot konnte weder optisch noch durch den Egger‐Test eine Asymmetrie detektiert werden, welche auf einen potenziellen Publikationsbias hindeuten könnte (p = 0.46) (Abbildung 2c).

### Initiale Heilungsrate

**ABBILDUNG 3:**
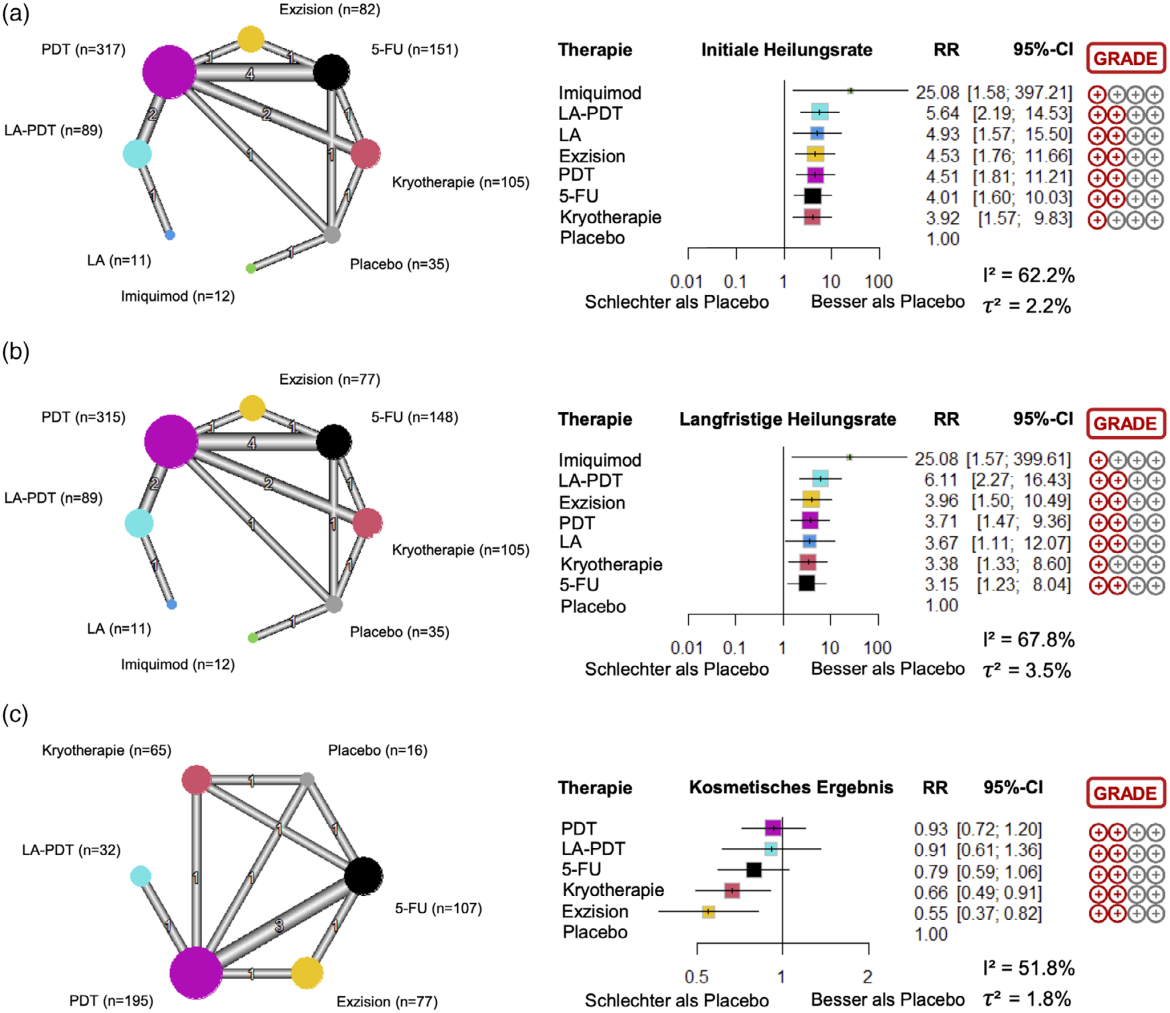
Netzwerkdiagramme und Forest‐Plots für alle untersuchten Endpunkte: (a) Läsionsspezifische initiale Heilungsrate (1–3 Monate nach Therapie), (b) läsionsspezifische langfristige Heilungsrate (6–12 Monate nach Therapie), und (c) kosmetisches Ergebnis. Das Risikoverhältnis (RR) verglichen mit Placebo war hierbei der Haupteffektschätzer. *Abk*.: 95%‐KI, 95% Konfidenzintervall; LA‐PDT, lasergestützte photodynamische Therapie; LA, Laserablation; PDT, photodynamische Therapie; 5‐FU, 5‐Fluoruracil.

Daten von neun Studien wurden in die NMA bezüglich des Endpunktes läsionsspezifische initiale Heilungsrate eingeschlossen. Insgesamt lieferten 16 Direktvergleich hierfür direkte Evidenz (Abbildung 3a). Alle Therapieoptionen zeigten signifikant bessere initiale Heilungsraten als Placebo. Imiquimod zeigte die höchste Heilungsrate (RR, 25.08; 95%‐KI, 1.58–397.21), wobei die Evidenz hierbei haupsächlich auf einer Studie beruht, welche Imiquimod mit Placebo verglichen hat.^37^ In dieser Studie erreichten elf von 15 Patienten, welche mit Imiquimod behandelt wurden, eine Heilung ihres MB, wohingegen kein Patient in der Placebogruppe eine Heilung erzielte. In unserer NMA folgten auf Imiquimod die Therapien mit LA‐PDT (5.64; 2.19–14.53), LA (4.93; 1.57–15.50), Exzision (4.53; 1.76–11.66), PDT (4.51; 1.81–11.21) und 5‐FU (4.01; 1.60–10.03). Kryotherapie zeigte die geringste Effektivität aller untersuchten Therapieoptionen (3.92; 1.57–9.83). Die Effektschätzer aller direkter und indirekter Vergleiche sind in  Tabelle 2 dargestellt. Nach GRADE war der Grad der Evidenz limitiert für alle Therapieoptionen. Die Studienheterogenität wurde als moderat eingestuft (I^2^ = 62.2%, τ^2^ = 2.2%).

**TABELLE 2:** Überblick über die direkte, indirekte sowie kombinierte Evidenz für jeden Vergleich auf Basis der Netzwerk‐Metaanalyse (NMA). Der Grad der Evidenz wurde anhand der für NMA modifizierten GRADE‐Richtlinien bewertet.

	DIREKTE EVIDENZ			INDIREKTE EVIDENZ		NMA	
Comparison	Anzahl Vergleiche	RR [95%‐KI]	Grad der Evidenz	RR [95%‐KI]	Grad der Evidenz	RR [95% CI]	Grad der Evidenz
*Initiale Heilungsrate*							
5‐FU vs. Kryotherapie	1	0,96 [0,68; 1,36]	●●○○^2^	1,15 [0,70; 1,89]	●●○○	1.02 [0.77; 1.36]	●●○○
5‐FU vs. Imiquimod	0	NA		0,16 [0,01; 2,94]	●○○○	0.16 [0.01; 2.94]	●○○○
5‐FU vs. LA	0	NA		0,81 [0,40; 1,67]	●●○○	0.81 [0.40; 1.67]	●●○○
5‐FU vs. LA‐PDT	0	NA		0,71 [0,52; 0,98]	●●○○	0.71 [0.52; 0.98]	●●○○
5‐FU vs. PDT	4	0,90 [0,74; 1,09]	●●○○^2^	0,39 [0,07; 2,01]	●●●○	0.89 [0.73; 1.08]	●●●○
5‐FU vs. Placebo	1	3,93 [1,55; 9,99]	●●○○^2^	7,14 [0,05; 1092,89]	●●○○	4.01 [1.60; 10.03]	●●○○
5‐FU vs. Exzision	1	0,96 [0,71; 1,29]	●●●○^1^	0,55 [0,27; 1,12]	●●○○	0.88 [0.67; 1.16]	●●●○
Kryotherapie vs. Imiquimod	0	NA		0,16 [0,01; 2,88]	●○○○	0.16 [0.01; 2.88]	●○○○
Kryotherapie vs. LA	0	NA		0,80 [0,38; 1,67]	●●○○	0.80 [0.38; 1.67]	●●○○
Kryotherapie vs. LA‐PDT	0	NA		0,70 [0,48; 1,00]	●●○○	0.70 [0.48; 1.00]	●●○○
Kryotherapie vs. PDT	2	0,86 [0,66; 1,13]	●●○○^2^	0,95 [0,42; 2,16]	●●○○	0.87 [0.67; 1.13]	●●○○
Kryotherapie vs. Placebo	1	4,08 [1,62; 10,25]	●●○○^2^	0,01 [0,00; 942,29]	●●○○	3.92 [1.57; 9.83]	●○○○^4^
Kryotherapie vs. Exzision	0	NA		0,87 [0,60; 1,24]	●●○○	0.87 [0.60; 1.24]	●●○○
Imiquimod vs. LA	0	NA		5,08 [0,26; 101,13]	●○○○	5.08 [0.26; 101.13]	●○○○
Imiquimod vs. LA‐PDT	0	NA		4,45 [0,24; 82,50]	●○○○	4.45 [0.24; 82.50]	●○○○
Imiquimod vs. PDT	0	NA		5,56 [0,30; 102,03]	●○○○	5.56 [0.30; 102.03]	●○○○
Imiquimod vs. Placebo	1	25,08 [1,58; 397,21]	●○○○^2^, ^3^	NE		25.08 [1.58; 397.21]	●○○○
Imiquimod vs. Exzision	0	NA		5,53 [0,30; 102,49]	●○○○	5.53 [0.30; 102.49]	●○○○
LA vs. LA‐PDT	1	0,87 [0,46; 1,67]	●●○○^1^, ^3^	NE		0.87 [0.46; 1.67]	●●○○
LA vs. PDT	0	NA		1,09 [0,55; 2,19]	●●○○	1.09 [0.55; 2.19]	●●○○
LA vs. Placebo	0	NA		4,93 [1,57; 15,50]	●●○○	4.93 [1.57; 15.50]	●●○○
LA vs. Exzision	0	NA		1,09 [0,52; 2,29]	●●○○	1.09 [0.52; 2.29]	●●○○
LA‐PDT vs. PDT	2	1,25 [0,97; 1,62]	●●●○^1^	NE		1.25 [0.97; 1.62]	●●○○
LA‐PDT vs. Placebo	0	NA		5,64 [2,19; 14,53]	●●○○	5.64 [2.19; 14.53]	●●○○
LA‐PDT vs. Exzision	0	NA		1,24 [0,85; 1,81]	●●●○	1.24 [0.85; 1.81]	●●●○
PDT vs. Placebo	1	4,41 [1,76; 11,05]	●●○○^2^	16,69 [0,01; 19130,66]	●●○○	4.51 [1.81; 11.21]	●●○○
PDT vs. Exzision	1	0,91 [0,67; 1,23]	●●●○^1^	1,57 [0,79; 3,13]	●●○○	0.99 [0.75; 1.31]	●●●○
Exzision vs. Placebo	0	NA		4,53 [1,76; 11,66]	●●○○	4.53 [1.76; 11.66]	●●○○
*Langfristige Heilungsrate*							
5‐FU vs. Kryotherapie	1	1,03 [0,66; 1,61]	●●○○^2^	0,84 [0,53; 1,32]	●●○○	0.93 [0.68; 1.28]	●●○○
5‐FU vs. Imiquimod	0	NA		0,13 [0,01; 2,33]	●○○○	0.13 [0.01; 2.33]	●○○○
5‐FU vs. LA	0	NA		0,86 [0,39; 1,89]	●●○○	0.86 [0.39; 1.89]	●●○○
5‐FU vs. LA‐PDT	0	NA		0,51 [0,34; 0,79]	●●○○	0.51 [0.34; 0.79]	●●○○
5‐FU vs. PDT	4	0,83 [0,65; 1,07]	●●○○^2^	1,41 [0,35; 6,60]	●●●○	0.85 [0.67; 1.08]	●●●○
5‐FU vs. Placebo	1	3,28 [1,25; 8,61]	●●○○^2^	1,63 [0,03; 79,74]	●●○○	3.15 [1.23; 8.04]	●●○○
5‐FU vs. Exzision	1	0,88 [0,62; 1,26]	●●●○^1^	0,44 [0,19; 1,04]	●●○○	0.79 [0.57; 1.10]	●●●○
Kryotherapie vs. Imiquimod	0	NA		0,13 [0,01; 2,50]	●○○○	0.13 [0.01; 2.50]	●○○○
Kryotherapie vs. LA	0	NA		0,92 [0,42; 2,04]	●●○○	0.92 [0.42; 2.04]	●●○○
Kryotherapie vs. LA‐PDT	0	NA		0,55 [0,36; 0,85]	●●○○	0.55 [0.36; 0.85]	●●○○
Kryotherapie vs. PDT	2	0,92 [0,71; 1,20]	●●○○^2^	0,76 [0,26; 2,24]	●●○○	0.91 [0.71; 1.17]	●●○○
Kryotherapie vs. Placebo	1	3,19 [1,24; 8,21]	●●○○^2^	26,25 [0,10; 6849,90]	●●○○	3.38 [1.33; 8.60]	●○○○^4^
Kryotherapie vs. Exzision	0	NA		0,85 [0,57; 1,28]	●●○○	0.85 [0.57; 1.28]	●●○○
Imiquimod vs. LA	0	NA		6,84 [0,34; 139,38]	●○○○	6.84 [0.34; 139.38]	●○○○
Imiquimod vs. LA‐PDT	0	NA		4,11 [0,22; 77,64]	●○○○	4.11 [0.22; 77.64]	●○○○
Imiquimod vs. PDT	0	NA		6,76 [0,36; 125,24]	●○○○	6.76 [0.36; 125.24]	●○○○
Imiquimod vs. Placebo	1	25,08 [1,57; 399,61]	●○○○^2^, ^3^	NE		25.08 [1.57; 399.61]	●○○○
Imiquimod vs. Exzision	0	NA		6,33 [0,34; 119,17]	●○○○	6.33 [0.34; 119.17]	●○○○
LA vs. LA‐PDT	1	0,60 [0,31; 1,17]	●●○○^1^, ^3^	NE		0.60 [0.31; 1.17]	●●○○
LA vs. PDT	0	NA		0,99 [0,47; 2,09]	●●○○	0.99 [0.47; 2.09]	●●○○
LA vs. Placebo	0	NA		3,67 [1,11; 12,07]	●●○○	3.67 [1.11; 12.07]	●●○○
LA vs. Exzision	0	NA		0,93 [0,41; 2,10]	●●○○	0.93 [0.41; 2.10]	●●○○
LA‐PDT vs. PDT	2	1,65 [1,16; 2,33]	●●●○^1^	NE		1.65 [1.16; 2.33]	●●○○
LA‐PDT vs. Placebo	0	NA		6,11 [2,27; 16,43]	●●○○	6.11 [2.27; 16.43]	●●○○
LA‐PDT vs. Exzision	0	NA		1,54 [0,96; 2,49]	●●●○	1.54 [0.96; 2.49]	●●●○
PDT vs. Placebo	1	3,81 [1,49; 9,75]	●●○○^2^	1,58 [0,01; 335,20]	●●○○	3.71 [1.47; 9.36]	●●○○
PDT vs. Exzision	1	0,84 [0,59; 1,21]	●●●○^1^	1,66 [0,72; 3,81]	●●○○	0.94 [0.67; 1.30]	●●●○
Exzision vs. Placebo	0	NA		3,96 [1,50; 10,49]	●●○○	3.96 [1.50; 10.49]	●●○○
*Kosmetisches Ergebnis*							
5‐FU vs. Kryotherapie	1	1,16 [0,78; 1,71]	●●○○^2^	1,30 [0,67; 2,51]	●●○○	1.19 [0.85; 1.67]	●●○○
5‐FU vs. LA‐PDT	0	NA		0,87 [0,60; 1,26]	●●○○	0.87 [0.60; 1.26]	●●○○
5‐FU vs. PDT	3	0,85 [0,69; 1,06]	●●○○^2^	NE		0.85 [0.69; 1.06]	●●○○
5‐FU vs. Placebo	1	0,77 [0,54; 1,09]	●●○○^2^	0,84 [0,51; 1,40]	●●○○	0.79 [0.59; 1.06]	●●○○
5‐FU vs. Exzision	1	1,54 [1,08; 2,19]	●●●○^1^	0,76 [0,25; 2,31]	●●○○	1.44 [1.03; 2.02]	●●●○
Kryotherapie vs. LA‐PDT	0	NA		0,73 [0,47; 1,13]	●●○○	0.73 [0.47; 1.13]	●●○○
Kryotherapie vs. PDT	1	0,71 [0,51; 0,97]	●●○○^2^	0,85 [0,27; 2,71]	●●○○	0.72 [0.53; 0.97]	●●○○
Kryotherapie vs. Placebo	1	0,66 [0,49; 0,91]	●●○○^2^	NE		0.66 [0.49; 0.91]	●●○○
Kryotherapie vs. Exzision	0	NA		1,21 [0,78; 1,88]	●●○○	1.21 [0.78; 1.88]	●●○○
LA‐PDT vs. PDT	1	0,98 [0,72; 1,33]	●●●○^1^	NE		0.98 [0.72; 1.33]	●●●○
LA‐PDT vs. Placebo	0	NA		0,91 [0,61; 1,36]	●●○○	0.91 [0.61; 1.36]	●●○○
LA‐PDT vs. Exzision	0	NA		1,66 [1,05; 2,61]	●●●○	1.66 [1.05; 2.61]	●●●○
PDT vs. Placebo	1	0,94 [0,72; 1,23]	●●○○^2^	0,83 [0,37; 1,86]	●●○○	0.93 [0.72; 1.20]	●●○○
PDT vs. Exzision	1	1,59 [1,12; 2,25]	●●●○^1^	3,27 [1,04; 10,28]	●●○○	1.69 [1.21; 2.36]	●●●○
Exzision vs. Placebo	0	NA		0,55 [0,37; 0,82]	●●○○	0.55 [0.37; 0.82]	●●○○

^1^ Moderates Verzerrungsrisiko (‐1),

^2^ hohes Verzerrungsrisiko (‐2),

^3^ Ungenauigkeit (großes Konfidenzintervall oder kleine Gruppengröße) (‐1),

^4^ Inkoheränz zwischen direkter und indirekter Evidenz (‐1). ●○○○, sehr gering; ●●○○, gering; ●●●○, moderat; ●●●●, hoch

*Abk*.: RR, Risikoverhältnis; 95%‐KI, 95%‐Konfidenzintervall; NA, nicht angegeben; NE, nicht einschätzbar; LA‐PDT, lasergestützte photodynamische Therapie; LA, Laserablation; PDT, photodynamische Therapie; 5‐FU, 5‐Fluoruracil

### Langfristige Heilungsrate

Neun Studien wurden bezüglich des Endpunktes läsionsspezifische langfristige Heilungsrate berücksichtigt. Hierbei lieferten ebenfalls 16 Direkvergleiche direkte Evidenz (Abbildung 3b). Alle Therapieoptionen erreichten signifikant bessere langfristige Heilungsraten als Placebo, wobei Imiquimod ebenfalls die höchste relative Effektstärke aufwies (25.08; 1.57–399.61). Die zweithöchste RR wurde von LA‐PDT (6.11; 2.27–16.43) erreicht, gefolgt von der Exzision (3.96; 1.50–10.49), PDT (3.71; 1.47–9.36), LA (3.67; 1.11–12.07), Kryotherapie (3.38; 1.33–8.60) und 5‐FU (3.15; 1.23–8.04). Der Grad der Evidenz war generell limitiert, und die Studienheterogenität war moderat (I^2^ = 67.8%, τ^2^ = 3.5%).

### Kosmetisches Ergebnis

Bezüglich des kosmetischen Ergebnissen, konnten nur vier Studien mit insgesamt elf Direktvergleichen analysiert werden (Abbildung 3c). Kryotherapie (0.66; 0.49–0.91) und Exzision (0.55; 0.37–0.82) zeigten signifikant geringere RR verglichen mit Placebo, was auf ein schlechteres kosmetisches Ergebnis hindeutet. Die verbleibenden Therapieoptionen waren statistisch Placebo nicht unterlegen (5‐FU: 0.79; 0.59–1.06; LA‐PDT: 0.91; 0.61–1.36; PDT: 0.93; 0.72–1.20). Der Grad der Evidenz war niedrig und die Studienheterogenität moderat (I^2^ = 51.8%, τ^2^ = 1.8%).

### Bewertung der Therapieoptionen

Die Therapieoptionen wurden anhand des minimal kontextualisierten Rahmenansatzes bewertet und entsprechend gereiht (Tabelle 3). Bezüglich der initialen Heilungsrate wurde LA‐PDT als beste Therapieoption bewertet, dicht gefolgt von der Exzision, LA und PDT. Kryotherapie und Imiquimod wurden als schlechteste Therapieoptionen bewertet. Hinsichtlich der langfristigen Heilungsrate, wurde die Exzision als beste Therapieoption bewertet, gefolgt von LA‐PDT und PDT, wohingegen Imiquimod und Kryotherapie erneut nach hinten gereiht wurden. PDT wurde bezüglich des kosmetischen Ergebnisses als beste Therapieoption bewertet, gefolgt von LA‐PDT und 5‐FU. Kryotherapie und Exzision wurden diesbezüglich am schlechtesten bewertet.

**TABELLE 3:** Ranking der Therapieoptionen für läsionsspezifische initiale Heilungsrate (1–3 Monate nach Therapie), läsionsspezifische langfristige Heilungsrate (6–12 Monate nach Therapie), und kosmetisches Ergebnis. Das Risikoverhältnis (RR) verglichen mit Placebo war der Haupteffektschätzer. Die Bewertung basiert auf dem minimal kontextualisierten Rahmenansatz für Netzwerk‐Metaanalysen, welcher die Effektschätzer, den Grad der Evidenz und die p‐Scores berücksichtigt.

Grad der Evidenz und Bewertung der Therapieoptionen	Rang	Intervention	RR [95%‐KI] verglichen mit Placebo	p‐Score
*Läsionsspezifische initiale Heilungsrate (1–3 Monate nach Therapie)*				
Geringer Evidenzgrad (⊕⊕◯◯)				
Alle Interventionen waren Placebo signifikant überlegen	1	LA‐PDT	5,64 [2,19; 14,53]	0,8931
	2	Exzision	4,53 [1,76; 11,66]	0,8038
	3	LA	4,93 [1,57; 15,50]	0,7982
	4	PDT	4,51 [1,81; 11,21]	0,5792
	5	5‐FU	4,01 [1,60; 10,03]	0,3240
Sehr geringer Evidenzgrad (⊕◯◯◯)				
Alle Interventionen waren Placebo signifikant überlegen	6	Kryotherapie	3,92 [1,57; 9,83]	0,3417
	7	Imiquimod	25,08 [1,58; 397,21]	0,2562
*Läsionsspezifische langfristige Heilungsrate (6–12 Monate nach Therapie)*				
Geringer Evidenzgrad (⊕⊕◯◯)				
Alle Interventionen waren Placebo signifikant überlegen	1	Exzision	3,96 [1,50; 10,49]	0,9121
	2	LA‐PDT	6,11 [2,27; 16,43]	0,8940
	3	PDT	3,71 [1,47; 9,36]	0,5782
	4	LA	3,67 [1,11; 12,07]	0,4288
	5	5‐FU	3,15 [1,23; 8,04]	0,3190
Sehr geringer Evidenzgrad (⊕◯◯◯)				
Alle Interventionen waren Placebo signifikant überlegen	6	Imiquimod	25,08 [1,57; 399,61]	0,4910
	7	Kryotherapie	3,38 [1,33; 8,60]	0,3342
*Kosmetisches Ergebnis*				
Geringer Evidenzgrad (⊕⊕◯◯)				
Kategorie 1: Placebo nicht signifikant unterlegen	1	PDT	0,93 [0,72; 1,20]	0,7440
	2	LA‐PDT	0,91 [0,61; 1,36]	0,6082
	3	5‐FU	0,79 [0,59; 1,06]	0,4078
Kategorie 2: Placebo signifikant unterlegen	4	Kryotherapie	0,66 [0,49; 0,91]	0,2089
	5	Exzision	0,55 [0,37; 0,82]	0,1813

*Abk*.: 95%‐KI, 95%‐Konfidenzintervall; LA‐PDT, lasergestützte photodynamische Therapie; LA, Laserablation; PDT, photodynamische Therapie; 5‐FU, 5‐Fluoruracil

### Unerwünschte Therapienebenwirkungen

**ABBILDUNG 4:**
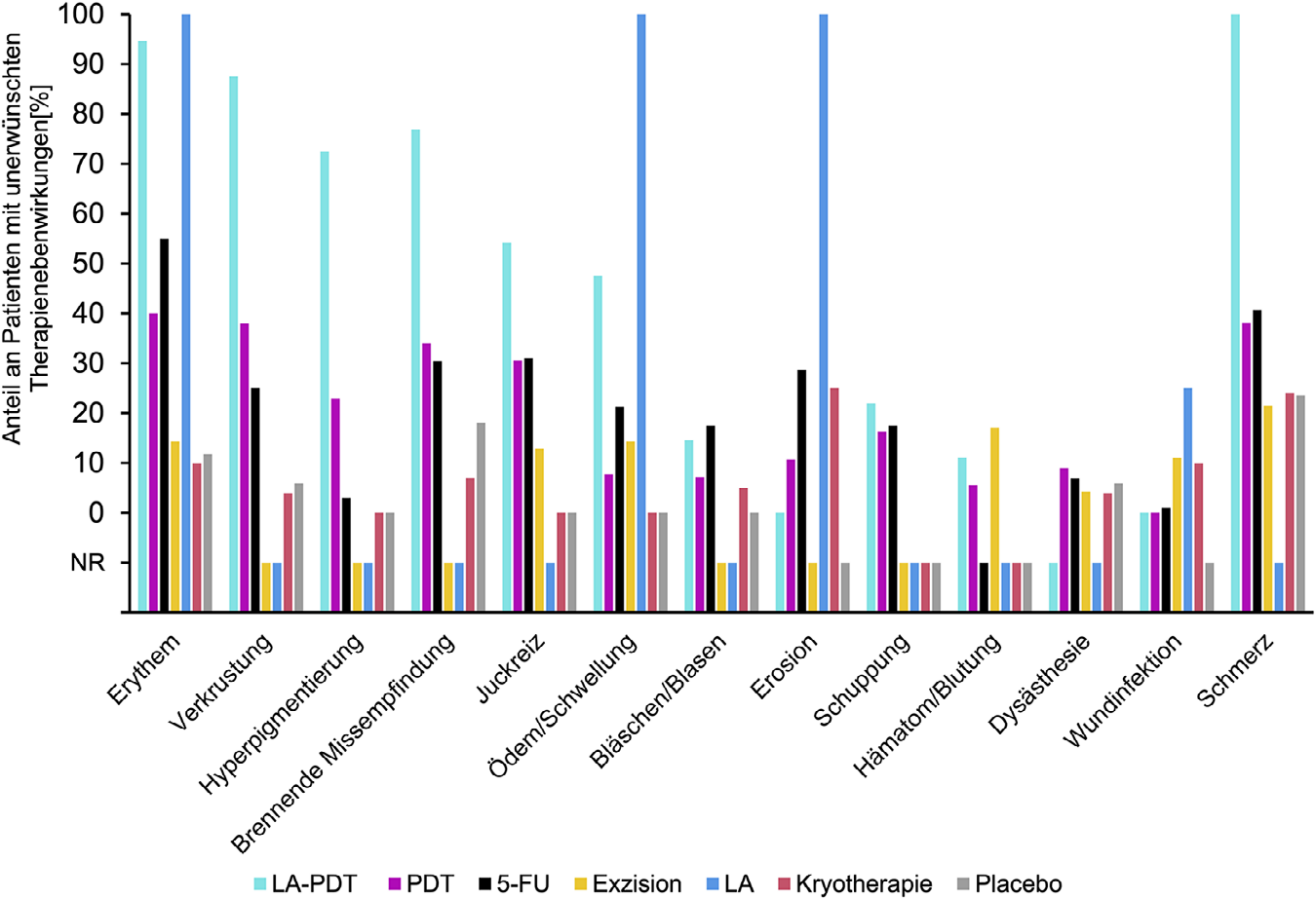
Das gruppierte Säulendiagramm zeigt für alle Therapieoptionen den Anteil an Patienten mit unerwünschten Therapienebenwirkungen. Für Imiquimod wurden keine Nebenwirkungen berichtet. Die Durchschnittswerte wurden nach den Gruppengrößen der einzelnen Studien gewichtet. *Abk*.: NB, nicht berichtet; LA‐PDT, lasergestütze photodynamische Therapie; LA, Laserablation; PDT, photodynamische Therapie; 5‐FU, 5‐Fluoruracil.

Das Auftreten von unerwünschten Therapienebenwirkungen war stark heterogen und wurde nicht regelhaft in den eingeschlossenen Studien berichtet, sodass eine qualitative Ergebnissynthese auf dem Boden einer NMA nicht möglich war. Ein Überblick über die berichteten Nebenwirkungen bietet Abbildung 4. Die Durchschnittswerte wurden anhand der Gruppengrößen der einzelnen Studien adjustiert (Tabelle S3, Online‐Supplement).

## DISKUSSION

Unsere NMA ist die erste umfassende Bewertung der relativen Heilungsraten verschiedener Therapieoptionen für MB, die in RCT untersucht wurden. Sie ermöglicht eine objektive Beurteilung auf Grundlage sowohl direkter als auch indirekter Vergleiche. Die Analyse liefert Erkenntnisse zu läsionsspezifischen initialen und langfristigen Heilungsraten sowie zum kosmetischen Ergebnis. Insgesamt wurden neun RCT mit 672 Patienten und 844 Läsionen zur qualitativen Datensynthese eingeschlossen.

In Bezug auf die Heilungsraten zeigten alle Therapieoptionen eine signifikant bessere Wirksamkeit im Vergleich mit Placebo, wobei Imiquimod die höchste relative Effektsärke sowohl für die initiale als auch die langfristige Heilungsrate aufwies. Hierbei ist jedoch zu beachten, dass nur eine einzige Studie, die eine signifikante Überlegenheit von Imiquimod gegenüber Placebo zeigte, einen direkten Vergleich zur NMA beitrug. Die Effektstärke von Imiquimod sollte mit Vorsicht interpretiert werden, jedoch zeigten mehrere nichtrandomisierte Studien ebenfalls vergleichbare Ansprechraten von bis zu 93%.^47^, ^48^, ^49^ Bezüglich der initialen Heilungsrate wurde die LA‐PDT am besten bewertet, gefolgt von Exzision, LA und konventioneller PDT. Diese Therapieoptionen zeigen somit konsistentere und verlässlichere initiale Heilungsraten verglichen mit topischen Therapieoptionen wie 5‐FU, Kryotherapie und Imiquimod. Diese Ergebnisse stehen im Einklang mit den Erkenntnissen zweier Übersichtsarbeiten, welche berichteten, dass PDT für die Behandlung des MB effektiver war als 5‐FU und Kryotherapie.^8^, ^9^ Eine systematische Übersichtsarbeit von Xue et al.^8^ zeigte außerdem, dass eine PDT in Kombination mit einem fraktionierten CO_2_‐Laser einer alleinigen PDT überlegen war. Diese Ergebnisse stützen unsere Bewertungsreihenfolge, wobei wir keine Studien finden konnten, die eine LA‐PDT direkt mit topischen Therapien wie 5‐FU, Kryotherapie oder Imiquimod verglichen hat. In Bezug auf die langfristige Heilungsrate wurden ähnliche Trends beobachtet. Während Imiquimod die höhere Effektstärke aufwies, bewerteten wir die Exzision als beste Therapieoption zur langfristigen Heilung. Es ist außerdem bekannt, dass die Exzision exzellente Langzeitheilungsraten aufweist.^50^ Im Gegensatz zu anderen ablativen oder topischen Verfahren wird die Läsion typischerweise mit einem Sicherheitsabstand oder unter mikrographischer Kontrolle entfernt. Verglichen mit der Exzision haben andere Therapien hierbei ein höheres Risiko, dass einzelne maligne Zellen nach der Therapie verbleiben. Dies kann zu späteren Rezidiven führen, auch wenn die Läsion inital geheilt erschien.

Das kosmetische Ergebnis ist ein wichtiger Faktor bei der Therapie des MB, insbesondere vor dem Hintergrund, dass viele Fälle in sichtbaren Arealen wie Kopf und Hals auftreten. Wir fanden, dass Kryotherapie und Exzision verglichen mit Placebo mit einem schlechteren kosmetischen Ergebnis verbunden waren. Im Gegensatz hierzu zeigten PDT, LA‐PDT und 5‐FU ein besseres kosmetisches Ergebnis, was sie zu attraktiven Therapieoptionen für Patienten macht, die neben der Effektivität auch großen Wert auf das kosmetische Ergebnis legen.

Unerwünschte Therapienebenwirkungen wurden von den eingeschlossenen Studien nur unregelmäßig berichtet, weshalb keine definitiven Aussagen zu den Sicherheitsprofilen der einzelnen Therapieoptionen getroffen werden konnten. Auf Grundlage der verfügbaren Daten zeigten sich jedoch keine signifikanten Sicherheitsbedenken, die eine der untersuchten Therapieoptionen aus der klinischen Praxis ausschließen würden. Ein besser standardisiertes Berichtsystem für unerwünschte Therapienebenwirkungen könnte zu einer besseren Nutzen‐Risiko‐Bewertung in zukünftigen klinischen Studien beitragen.

Ein wesentlicher Vorteil der NMA besteht darin, dass sie die Integration von Daten aus einer Vielzahl von Studien ermöglicht und so den Vergleich unterschiedlicher Therapieoptionen erlaubt, die in RCT nicht direkt gegeneinander getestet wurden. Allerdings war die Gesamtqualität der Evidenz durch die geringe Anzahl direkter Vergleiche sowie die Heterogenität der Studien, bedingt durch Unterschiede in den Patientenkollektiven, Studiendesigns, Therapien und Endpunkten, eingeschränkt. Beispielsweise gibt es, im Gegensatz zu AK,^51^ für MB keine standardisierten Endpunktparameter, sodass wir uns auf die läsionsspezifischen Heilungsraten fokussieren mussten. Viele der eingeschlossenen Studien wiesen außerdem ein hohes Verzerrungsrisiko auf, besonders in Bezug auf den Randomisierungsprozess, wodurch der Grad der Evidenz limitiert ist. Außerdem berichteten nur drei Studien von einer adäquaten Fallzahlberechnung, wodurch die Verlässlichkeit und Übertragbarkeit der Ergebnisse eingeschränkt sein könnte. Zusätzlich war der Zeitraum der eingeschlossenen Studien sehr lang (1996–2024), was Unterschiede in Methodik, diagnostischen Kriterien und Auswertungsmethoden im Laufe der Zeit als zusätzliches Verzerrungsrisiko hinzufügt. Diese Faktoren tragen zur Heterogenität bei und sollten bei der Bewertung der Ergebnisse berücksichtigt werden. Ein zusätzliches Verzerrungsrisiko ergibt sich zudem aus der Kombination von ALA‐PDT und MAL‐PDT sowie verschiedener ablativer Lasersysteme zur besseren Vergleichbarkeit und um kleine unverbundene Subnetzwerke zu vermeiden. Eine Studie schloss außerdem immunsupprimierte Patienten ein, was zu einem zusätzlichen Verzerrungsrisko führen könnte. Im adjustierten Funnelplot zeigte sich keine Asymmetrie, jedoch ist die Aussagekraft des Funnelplots und des Egger‐Tests aufgrund der geringen Anzahl an Studien limitiert. Auf Grundlage der aktuellen Studienlandschaft konnten wir jedoch keine Hinweise auf einen Publikationsbias identifizieren.

Zusammenfassend liefert unsere NMA einen umfassenden Vergleich unterschiedlicher Therapieoptionen für MB und bietet somit eine evidenzbasierte Grundlage für klinische Therapieentscheidungen. Die photodynamische Therapie mit topischer Applikation von Aminolävulinsäure sowie die PDT erzielten sehr gute Heilungsraten bei gleichzeitig gutem kosmetischem Ergebnis. Die chirurgische Exzision stellt jedoch trotz ihres ungünstigeren kosmetischen Ergebnisses aufgrund der langfristigen Heilungsraten die Therapie der Wahl dar. Die Ergebnisse unterstreichen zudem die Notwendigkeit weiterer standardisierter Studien, um die langfristige Wirksamkeit, Sicherheit und kosmetischen Ergebnisse der Behandlungen von MB besser bewerten zu können.

## DANKSAGUNG

Open access Veröffentlichung ermöglicht und organisiert durch Projekt DEAL.

## INTERESSENKONFLIKT

Keiner.

## Supporting information



Supplementary information

Supplementary information
