# Supplementary material for: Therapien des Morbus Bowen: Systematisches Review und Netzwerk‐Metaanalyse randomisierter kontrollierter Studien
Source: J Dtsch Dermatol Ges. 2025 Nov 14;23(11):1373–86. [Article in German] doi: 10.1111/ddg.15866_g (PMC12619028; doi:10.1111/ddg.15866_g)
Supplement: Supplementary file 1 — Supplementary information [file DDG-23-1373-s002.docx]

**Tabelle S1**

| # | Suchbegriffe |
| --- | --- |
| 1 | Morbus Bowen.mp. |
| 2 | bowen.mp. |
| 3 | Bowen's disease.mp. |
| 4 | squamous cell carcinoma in situ.mp. |
| 5 | laser.mp. or laser/ |
| 6 | PDT.mp. |
| 7 | photodynamic therapy.mp. |
| 8 | imiquimod.mp. |
| 9 | cryo.mp. |
| 10 | cryotherapy.mp. |
| 11 | cryosurgery.mp. |
| 12 | 5-FU.mp. |
| 13 | 5-fluoruracil.mp. |
| 14 | fluoruracil.mp. |
| 15 | mohs.mp. |
| 16 | 1 or 2 or 3 or 4 |
| 17 | 5 or 6 or 7 or 8 or 9 or 10 or 11 or 12 or 13 or 14 or 15 |
| 18 | 16 and 17 |

Tabelle S2

| Autor | Intervention | Grund für Ausschluss | Beschreibung |
| --- | --- | --- | --- |
| de Haas 2007 | ALA-PDT mit einfacher Beleuchtung vs. ALA-PDT mit doppelter Beleuchtung | Falsche Intervention | Wir haben nicht zwischen einmaliger und zweimaliger Beleuchtung unterschieden, um kleine und unverbundene Subnetzwerke zu vermeiden |
| Puizina-Ivic 2008 | ALA-PDT mit einfacher Beleuchtung vs. ALA-PDT mit doppelter Beleuchtung | Falsche Intervention | Wir haben nicht zwischen einmaliger und zweimaliger Beleuchtung unterschieden, um kleine und unverbundene Subnetzwerke zu vermeiden |
| Morton 2000 | ALA-PDT Rotlicht vs. ALA-PDT Grünlicht | Falsche Intervention | Wir haben nicht zwischen Rot- und Grünlicht unterschieden, um kleine und unverbundene Subnetzwerke zu vermeiden |
| Lui 2004 | Intravenöses Verteporfin mit Rotlicht bei 60 vs. 120 vs. 180 J/cm^2^ | Falsche Intervention | Wir haben nicht zwischen unterschiedlichen Energiedichten (Fluence) unterschieden, um kleine und unverbundene Subnetzwerke zu vermeiden |
| Ellen 2007 | ALA-PDT mit einfacher Beleuchtung vs. ALA-PDT mit doppelter Beleuchtung | Falsche Intervention | Wir haben nicht zwischen einmaliger und zweimaliger Beleuchtung unterschieden, um kleine und unverbundene Subnetzwerke zu vermeiden |
| Genouw 2018 | Fraktionierter CO_2_-Laser vs. kontinuierlicher CO_2_-Laser | Falsche Intervention | Wir haben nicht zwischen fraktioniertem und kontinuierlichem Laser unterschieden, um kleine und unverbundene Subnetzwerke zu vermeiden |
| Wu 2018 | ALA-PDT vs. ALA-PDT nach Mikronadelung | Falsche Intervention | Wir haben die Mikronadelung ausgeschlossen, da sie keine etablierte Behandlung für Morbus Bowen ist und um kleine und unverbundene Subnetzwerke zu vermeiden |
| Morton 2005 | MAL-PDT vs. Kryotherapie vs. 5-FU vs. Placebo | Duplikat | Der Titel wurde bereits in unsere Analyse inkludiert (Morton 2006) |
| Morton 2004 | MAL-PDT vs. Kryotherapie vs. 5-FU vs. Placebo | Duplikat | Der Titel wurde bereits in unsere Analyse inkludiert (Morton 2006) |
| Ibbotson 2022 | Niedrigintensive ALA-PDT vs. konventionelle ALA-PDT | Falsche Intervention | Wir haben nicht zwischen niedrigintensiver und konventioneller ALA-PDT unterschieden, um kleine und unverbundene Subnetzwerke zu vermeiden |
| Mizutani 2012 | ALA-PDT mit Excimer-Laser vs. PDT mit Metallhalogenlampe | Falsche Intervention | Wir haben nicht zwischen ALA-PDT mit Excimer-Laser und Metallhalogenlampe unterschieden, um kleine und unverbundene Subnetzwerke zu vermeiden |
| NCT00384124 | Topisches Imiquimod vs. Placebo | Duplikat | Der Titel wurde bereits in unsere Analyse inkludiert (Patel 2006) |
| NCT03909646 | Exzision vs. MAL-PDT vs. 5-FU | Duplikat | Der Titel wurde bereits in unsere Analyse inkludiert (Ahmady 2024) |
| NCT03320447 | Er:YAG-Laser gestützte MAL-PDT vs. MAL-PDT | Duplikat | Der Titel wurde bereits in unsere Analyse inkludiert (Kim 2018) |
| NCT03012009 | CO_2_-Laser gestützte MAL-PDT vs. MAL-PDT | Keine Daten verfügbar | Die Studie ist geschlossen, letztes Update am 25.01.2018, keine Daten verfügbar |
| 2005-005171-14 | MAL-PDT vs. ALA-PDT | Falscher Endpunkt | Die Studie wurde aufgrund des falschen Endpunktes ausgeschlossen (Schmerz) |

**Tabelle S3**

| Therapie | Studie | Unerwünschte Therapienebenwirkung | | | | | | | | | | | | | | | | | | | | | | | | | | | | | | | | | | | | | | |
| --- | --- | --- | --- | --- | --- | --- | --- | --- | --- | --- | --- | --- | --- | --- | --- | --- | --- | --- | --- | --- | --- | --- | --- | --- | --- | --- | --- | --- | --- | --- | --- | --- | --- | --- | --- | --- | --- | --- | --- | --- |
|  |  | **Erythem** | | | **Verkrustung** | | | **Hyperpigmentierung** | | | **Brennende Missempfindung** | | | **Juckreiz** | | | **Ödem/Schwellung** | | | **Bläschen/Blasen** | | | **Erosion** | | | **Schuppung** | | | **Hämatom/Blutung** | | | **Dysästhesie** | | | **Wundinfektion** | | | **Schmerz** | | |
|  |  | **UAW** | **Total** | **[ %]** | **UAW** | **Total** | **[ %]** | **UAW** | **Total** | **[ %]** | **UAW** | **Total** | **[ %]** | **UAW** | **Total** | **[ %]** | **UAW** | **Total** | **[ %]** | **UAW** | **Total** | **[ %]** | **UAW** | **Total** | **[ %]** | **UAW** | **Total** | **[ %]** | **UAW** | **Total** | **[ %]** | **UAW** | **Total** | **[ %]** | **UAW** | **Total** | **[ %]** | **UAW** | **Total** | **[ %]** |
| LA-PDT | Kim 2018 | 28 | 30 | 93 | 24 | 30 | 80 | 23 | 30 | 77 | 22 | 30 | 73 | 21 | 30 | 70 | 9 | 30 | 30 | 3 | 30 | 10 |  |  |  |  |  |  |  |  |  |  |  |  |  |  |  |  |  |  |
|  | Cai 2015 | 10 | 10 | 100 |  |  |  |  |  |  |  |  |  |  |  |  | 10 | 10 | 100 |  |  |  |  |  |  |  |  |  |  |  |  |  |  |  |  |  |  |  |  |  |
|  | Ko 2014 | 17 | 18 | 94 | 18 | 18 | 100 | 12 | 18 | 67 | 15 | 18 | 83 | 5 | 18 | 28 |  |  |  | 4 | 18 | 22 | 0 | 18 | 0 | 4 | 18 | 22 | 2 | 18 | 11 |  |  |  | 0 | 18 | 0 | 18 | 18 | 100 |
|  | Total | 55 | 58 | 95 | 42 | 48 | 88 | 35 | 48 | 73 | 37 | 48 | 77 | 26 | 48 | 54 | 19 | 40 | 48 | 7 | 48 | 15 | 0 | 18 | 0 | 4 | 18 | 22 | 2 | 18 | 11 |  |  |  | 0 | 18 | 0 | 18 | 18 | 100 |
| PDT | Kim 2018 | 27 | 30 | 90 | 24 | 30 | 80 | 21 | 30 | 70 | 20 | 30 | 67 | 20 | 30 | 67 | 7 | 30 | 23 | 2 | 33 | 6 |  |  |  |  |  |  |  |  |  |  |  |  |  |  |  |  |  |  |
|  | Ahmady 2024 | 37 | 75 | 49 | 28 | 75 | 37 |  |  |  |  |  |  | 13 | 75 | 17 | 7 | 75 | 9 | 5 | 75 | 7 | 8 | 75 | 11 | 12 | 75 | 16 |  |  |  |  |  |  |  |  |  | 23 | 75 | 31 |
|  | Ko 2014 | 16 | 18 | 89 | 18 | 18 | 100 | 10 | 18 | 56 | 13 | 18 | 72 | 4 | 18 | 22 |  |  |  | 2 | 18 | 11 |  |  |  | 3 | 18 | 17 | 1 | 18 | 6 |  |  |  | 0 | 18 | 0 | 18 | 18 | 100 |
|  | Morton 2006 | 8 | 96 | 8 | 8 | 96 | 8 | 3 | 96 | 3 | 16 | 96 | 17 |  |  |  | 2 | 96 | 2 |  |  |  |  |  |  |  |  |  |  |  |  | 9 | 96 | 9 |  |  |  | 19 | 95 | 20 |
|  | Perrett 2007 |  |  |  | 8 | 8 | 100 | 1 | 8 | 13 |  |  |  | 3 | 8 | 38 |  |  |  |  |  |  |  |  |  |  |  |  |  |  |  |  |  |  |  |  |  | 8 | 8 | 100 |
|  | Salim 2003 |  |  |  |  |  |  |  |  |  |  |  |  |  |  |  |  |  |  |  |  |  |  |  |  |  |  |  |  |  |  |  |  |  |  |  |  | 14 | 19 | 74 |
|  | Morton 1996 |  |  |  |  |  |  |  |  |  |  |  |  |  |  |  |  |  |  |  |  |  |  |  |  |  |  |  |  |  |  |  |  |  |  |  |  |  |  |  |
|  | Total | 88 | 219 | 40 | 86 | 227 | 38 | 35 | 152 | 23 | 49 | 144 | 34 | 40 | 131 | 31 | 16 | 201 | 8 | 9 | 126 | 7 | 8 | 75 | 11 | 15 | 93 | 16 | 1 | 18 | 6 | 9 | 96 | 9 | 0 | 18 | 0 | 82 | 215 | 38 |
| 5-FU | Ahmady 2024 | 50 | 80 | 63 | 23 | 80 | 29 |  |  |  | 31 | 80 | 39 | 29 | 80 | 36 | 17 | 80 | 21 | 14 | 80 | 18 | 23 | 80 | 29 | 14 | 80 | 18 |  |  |  |  |  |  | 1 | 80 | 1 | 31 | 80 | 39 |
|  | Morton 2006 | 10 | 30 | 33 | 4 | 30 | 13 | 1 | 30 | 3 | 2 | 30 | 7 | 5 | 30 | 17 |  |  |  |  |  |  |  |  |  |  |  |  |  |  |  | 2 | 30 | 7 |  |  |  | 10 | 30 | 33 |
|  | Salim 2003 |  |  |  |  |  |  |  |  |  |  |  |  |  |  |  |  |  |  |  |  |  |  |  |  |  |  |  |  |  |  |  |  |  |  |  |  | 10 | 15 | 67 |
|  | Total | 60 | 110 | 55 | 27 | 110 | 25 | 1 | 30 | 3 | 33 | 110 | 30 | 34 | 110 | 31 | 17 | 80 | 21 | 14 | 80 | 18 | 23 | 80 | 29 | 14 | 80 | 18 |  |  |  | 2 | 30 | 7 | 1 | 80 | 1 | 51 | 125 | 41 |
| Exzision | Ahmady 2024 | 10 | 70 | 14 |  |  |  |  |  |  |  |  |  | 9 | 70 | 13 | 10 | 70 | 14 |  |  |  |  |  |  |  |  |  | 12 | 70 | 17 | 3 | 70 | 4 | 8 | 70 | 11 | 15 | 70 | 21 |
|  | Total | 10 | 70 | 14 |  |  |  |  |  |  |  |  |  | 9 | 70 | 13 | 10 | 70 | 14 |  |  |  |  |  |  |  |  |  | 12 | 70 | 17 | 3 | 70 | 4 | 8 | 70 | 11 | 15 | 70 | 21 |
| LA | Cai 2015 | 8 | 8 | 100 |  |  |  |  |  |  |  |  |  |  |  |  | 8 | 8 | 100 |  |  |  | 8 | 8 | 100 |  |  |  |  |  |  |  |  |  | 2 | 8 | 25 |  |  |  |
|  | Total | 8 | 8 | 100 |  |  |  |  |  |  |  |  |  |  |  |  | 8 | 8 | 100 |  |  |  | 8 | 8 | 100 |  |  |  |  |  |  |  |  |  | 2 | 8 | 25 |  |  |  |
| Placebo | Morton 2006 | 2 | 17 | 12 | 1 | 17 | 6 | 0 | 17 | 0 | 3 | 17 | 18 | 0 | 17 | 0 | 0 | 17 | 0 | 0 | 17 | 0 |  |  |  |  |  |  |  |  |  | 1 | 17 | 6 |  |  |  | 4 | 17 | 24 |
|  | Total | 2 | 17 | 12 | 1 | 17 | 6 | 0 | 17 | 0 | 3 | 17 | 18 | 0 | 17 | 0 | 0 | 17 | 0 | 0 | 17 | 0 |  |  |  |  |  |  |  |  |  | 1 | 17 | 6 |  |  |  | 4 | 17 | 24 |
| Kryotherapie | Morton 2006 | 8 | 82 | 10 | 3 | 82 | 4 | 0 | 82 | 0 | 6 | 82 | 7 | 0 | 82 | 0 | 0 | 82 | 0 | 4 | 82 | 5 |  |  |  |  |  |  |  |  |  | 3 | 82 | 4 |  |  |  | 20 | 82 | 24 |
|  | Morton 1996 |  |  |  |  |  |  |  |  |  |  |  |  |  |  |  |  |  |  |  |  |  | 5 | 20 | 25 |  |  |  |  |  |  |  |  |  | 2 | 20 | 10 |  |  |  |
|  | Total | 8 | 82 | 10 | 3 | 82 | 4 | 0 | 82 | 0 | 6 | 82 | 7 | 0 | 82 | 0 | 0 | 82 | 0 | 4 | 82 | 5 | 5 | 20 | 25 |  |  |  |  |  |  | 3 | 82 | 4 | 2 | 20 | 10 | 20 | 82 | 24 |

*Abk.:* UAW, unerwünschte Arzneimittelwirkung; PDT, photodynamische Therapie; LA-PDT, laserassistierte photodynamische Therapie; 5-FU, 5-Fluoruracil
